# Propidium Monoazide Integrated With qPCR Enables Rapid and Universal Detection of Infectious Porcine Reproductive and Respiratory Syndrome Viruses

**DOI:** 10.1155/tbed/6250851

**Published:** 2024-12-21

**Authors:** Wenhao Qi, Yuejia Qiu, Dashi Zhao, Ming Qiu, Hong Lin, Meng Cui, Shuai Yang, Wanglong Zheng, Jianzhong Zhu, Nanhua Chen

**Affiliations:** ^1^Department of Preventive Veterinary Medicine, College of Veterinary Medicine, Yangzhou University, Yangzhou 225009, Jiangsu, China; ^2^Joint International Research Laboratory of Agriculture and Agri-Product Safety, Yangzhou University, Yangzhou 225009, Jiangsu, China; ^3^International Research Laboratory of Prevention and Control of Important Animal Infectious Diseases and Zoonotic Diseases of Jiangsu Higher Education Institutions, Yangzhou University, Yangzhou 225009, Jiangsu, China; ^4^Comparative Medicine Research Institute, Yangzhou University, Yangzhou 225009, Jiangsu, China; ^5^Jiangsu Co-Innovation Center for Prevention and Control of Important Animal Infectious Diseases and Zoonoses, Yangzhou University, Yangzhou 225009, Jiangsu, China; ^6^Key Laboratory of Animal Pathogen Infection and Immunology of Fujian Province, Fujian Agriculture and Forestry University, Fuzhou 350002, Fujian, China

**Keywords:** application, infectious virus detection, porcine reproductive and respiratory syndrome virus (PRRSV), propidium monoazide qPCR (PMA-qPCR)

## Abstract

Infectious porcine reproductive and respiratory syndrome virus (PRRSV) causes PRRS, but noninfectious PRRSV cannot. PCR and ELISA are commonly used for PRRSV detection but they cannot discriminate PRRSV infectivity. Virus isolation is a gold standard to determine virus infectivity. However, it is time-consuming. Therefore, we developed a propidium monoazide (PMA) qPCR assay for rapid and universal detection of infectious PRRSV in this study. After comparing the inactivation efficacies of distinct disinfectants, ultraviolet (UV) light, and heat, heat at 72°C for 15 min was determined as an effective strategy for PRRSV inactivation, which was confirmed by virus isolation and immunofluorescence assay (IFA) detection. In addition, PMA pretreatment parameters were optimized, including PMA concentration (5 μM), PMA binding time (25 min), PMA binding temperature (37°C), and photolysis time (25 min). The optimal concentration of primers and probes adapted from our previous study was redetermined. The optimized PMA-qPCR assay exhibited satisfied specificity, sensitivity, and reproducibility. Furthermore, the new PMA-qPCR was applied on the detection of 270 clinical samples (including 57 environmental feces, 177 lungs, 33 lymph nodes [LN], and 3 sera) and compared with previously developed qPCR. Eighty samples were qPCR positive, while only 63 samples were PMA-qPCR positive. No virus could be isolated in the 17 qPCR-positive but PMA-qPCR-negative clinical samples; meanwhile, PRRSV could be isolated in representative PMA-qPCR-positive samples, supporting that only live PRRSV isolates in distinct samples could be detected by this PMA-qPCR assay. In conclusion, this study provides the first PMA-qPCR assay for rapid and universal detection of infectious PRRSV, offering an alternative and effective method for PRRSV diagnosis, prevention, and control.

## 1. Introduction

Porcine reproductive and respiratory syndrome (PRRS) is a highly contagious infectious disease jeopardizing the global swine industry. The etiological agent, PRRS virus (PRRSV) is an enveloped, positive-sense, single-stranded RNA virus belonging to the genus *Betaarterivirus* in the family *Arteriviridae* [1]. PRRSV is one of the most rapidly evolving RNA viruses, which was first isolated in the Netherlands in 1991 and subsequently in the United States [2, 3]. PRRSV isolates were previously divided into two genotypes (European and North American types) and are currently clustered into two species (PRRSV-1 and PRRSV-2) [4, 5].

In China, classical PRRSV-2 (CH-1a-like) was first isolated in 1995 [6]. Highly pathogenic PRRSV-2 (HP-PRRSV-2, JXA1-like) causing severe outbreaks was identified in 2006 [7]. Since 2013, NADC30-like PRRSV-2 isolates became predominant [8]. Subsequently, NADC34-like PRRSV-2 isolates became prevalent from 2017 [9]. Meanwhile, wild-type PRRSV-1 strains (BJEU06-1 and NMEU09-1) were also isolated in mainland China from 2006 [10], which have been detected in at least 23 provinces [11]. Therefore, it's extremely difficult to diagnose, prevent, and control PRRS in China due to the co-existence of diverse PRRSV-2 and PRRSV-1 isolates.

Effective diagnosis is the first step for PRRS control. Several methods have been used for PRRSV detection, including virus isolation, serological methods, and molecular biological assays. Virus isolation can determine live viruses, but it is time-consuming, high-cost, and complicated. Serological methods such as ELISA detect PRRSV specific antibodies, which cannot distinguish whether the antibodies were induced by live virus infection or kill vaccine immunization. Molecular biological assays (both RT-PCR and real-time RT-PCR) that targeted on viral nucleic acids are the most widely used in clinics [12, 13], but they cannot identify whether the viral RNA is from an infectious virus or not. Therefore, novel assays are needed for evaluating PRRSV infectivity to avoid misdiagnoses on environmental contamination situations and pig healthy status.

Propidium monoazide (PMA) is a photo-reactive double-stranded nucleic acid intercalating dye that covalently binds to DNA/RNA upon exposure to intense visible light and then to prevent the DNA/RNA from being amplified by PCR [14]. When live microorganisms with intact cell membranes are exposed to PMA and light, the dye cannot penetrate the cell membrane. Thus no DNA/RNA modification occurs, which can still be amplified by PCR [15]. Accordingly, PMA pretreatment along with PCR has been utilized to distinguish not only live and dead bacteria but also infectious and noninfectious enveloped viruses [16–20]. However, no corresponding assay has been developed to detect PRRSV infectivity yet.

In the present study, we evaluated whether PMA pretreatment could be coupled with qPCR (PMA-qPCR) to discriminate viral nucleic acid from infectious and noninfectious PRRSV and determine its infectivity. PRRSV disinfection strategies, PMA pretreatment parameters, and PMA-qPCR amplification conditions were optimized. In addition, the efficacy of this new PRRSV PMA-qPCR method was evaluated by comparing it with our previously developed PRRSV qPCR assay in the detection of 270 clinical samples collected from 2020 to 2024.

## 2. Materials and Methods

### 2.1. Viruses and Cells

Representative PRRSV strains utilized in this study were all isolated and stored in our laboratory, which include PRRSV-2 NADC30-like SD17-38 isolate and NADC34-like Anheal-1 isolate (lineage 1), PRRSV-2 VR-2332-like JSYC20-05-1 isolate (lineage 5), CH-1a-like SD1612-1 isolate and HP-PRRSV-2 XJ17-5 isolate (lineage 8), and PRRSV-1 SD1291 isolate [21–23]. The other viruses used for PMA-qPCR specificity evaluation include porcine epidemic diarrhea virus (PEDV) XM1-2 strain, porcine deltacoronavirus (PDCoV) SDLY2302-1721 strain, classical swine fever virus (CSFV) JS1805-2 strain, pseudorabies virus (PRV) XJ03 strain, porcine circovirus 2 (PCV2) SD17-36 strain porcine parvovirus 7 (PPV7) JSYZ20190725-717 strain [24–26]. Pulmonary alveolar macrophages (PAMs) were maintained in Roswell Park Memorial Institute 1640 medium (RPMI-1640) (HyClone, USA) supplemented with 10% fetal bovine serum (FBS) (EallBio, China), 100 U/ml penicillin and 100 μg/ml streptomycin (Solarbio, China). Marc-145 cells were cultured in Dulbecco minimum essential medium (DMEM) (HyClone, USA) containing 10% FBS and antibiotics [27].

### 2.2. Clinical Sample Information

A total of 270 clinical samples, including 57 environmental feces, 177 lungs, 33 lymph nodes (LN), and 3 sera, were collected from nine cities/provinces (Beijing, Shandong, Henan, Sichuan, Guangdong, Fujian, Zhejiang, Heilongjiang and Jiangsu) of China from April 2020 to February 2024. All clinical samples were detected by both qPCR and PMA-qPCR assays.

### 2.3. Inactivating Treatments

Virus inactivation was executed by heat, ultraviolet (UV) light, and three disinfectants, including parachlormetaxylenol (PCMX) (Tianjin Zhonglian Chemical Reagent Co. Ltd), bromogeramine (Jiangxi Sarcandra Glabra Disinfection Products Co. Ltd), and bleach (Dezhou Chengze Disinfection Technology Co. Ltd). The same amount of PRRSV (200 μl 10^3^ TCID_50_/ml XJ17-5 strain) was used for all inactivation evaluations. To evaluate inactivation efficacy by heat, PRRSV was treated at 56, 64, 72, and 80°C for various times (5, 10, 15, and 20 min), respectively. To evaluate inactivation efficacy by UV light, PRRSV was disinfected for 10, 20, and 30 min using a low-pressure mercury vapor lamp emitting monochromatic (254 nm) UV light (Qingdao Haier Biomedical Co. Ltd). To evaluate inactivation efficacy by disinfectants, 50 μl distinct concentrations of PCMX (2, 4, 10 g/l), bromogeramine (2, 3, 4 g/l), and bleach (300, 500 mg/l) were added to 200 μl PRRSV sample for 30 min, respectively. All the final concentrations of disinfection reagents were set according to product recommendations. Each sample was prepared in triplex.

### 2.4. Optimization of PMA treatment

To determine PMA pretreatment parameters for discriminating inactivated PRRSV, inactivated PRRSV was pretreated with different concentrations (1, 5, 10, 25, 50, 100 μM) of PMA (Biotium, United States) dissolved in 20% DMSO in dark for various times (5, 10, 15, 20, 25 min) at different temperatures (0, 20, 37°C). Subsequently, samples were subjected to photolysis at blue light (BL-12, Beijing Labgic Technology Co. Ltd) for 5, 10, 15, 20, and 25 min. Subsequently, samples were submitted to viral RNA extraction using TRIpure Reagent (Aidlab, Beijing, China). The first-strand cDNA was synthesized using the PrimeScript 1st Strand cDNA Synthesis Kit (TaKaRa, Japan).

### 2.5. PMA-qPCR Amplification

One probe and two primers used for RRRSV PMA-qPCR were adapted from the universal PRRSV qPCR assay developed in our previous study [25]. The concentrations of primers and probe were further optimized to yield the lowest cycle threshold (Ct) for live virus and the highest Ct for inactivated virus in PMA-qPCR detection. Premix Ex Taq (Probe qPCR, 2x) (TaKaRa, Japan) and StepOne Plus Real-Time PCR System (Thermo Fisher Scientific) were used in both PMA-qPCR and qPCR detection.

### 2.6. Immunofluorescence Assay (IFA)

IFA was performed in both PAMs and Marc-145 cells [28]. PAMs were prepared from lung lavage fluid of 6-week-old PRRSV-free healthy piglets [29]. The infected PAMs and Marc-145 cells were collected at 72 hpi for IFA detection. Briefly, the infected cells were washed thrice with PBS and fixed using 4% paraformaldehyde. Cells were permeabilized with 0.5% TritonX-100 for 10 min and blocked with 1% BSA for 2 h. PRRSV-specific murine mAb 15A1 (1:500) against N protein was utilized as the primary antibody, while Dylight 594 goat anti-mouse IgG (1:1000, Invitrogen, USA) was used as the secondary antibody [21]. Cellular nuclei were counterstained using 4′,6-diamidino-2-phenylindole (DAPI). The cells were visualized by laser scanning confocal microscope (LSCM, Leica SP8, Solms, Germany).

### 2.7. Statistical Analysis

The data of *Δ*Ct values and viral RNA levels in this study were shown as means ± standard deviations (SD). The statistical analysis was performed using the Mann–Whitney *U* test embedded in the GraphPad Prism 8 XML project [30].

## 3. Results

### 3.1. PRRSV Inactivation by Disinfectants, UV Light, and Heat

**Figure 1:**
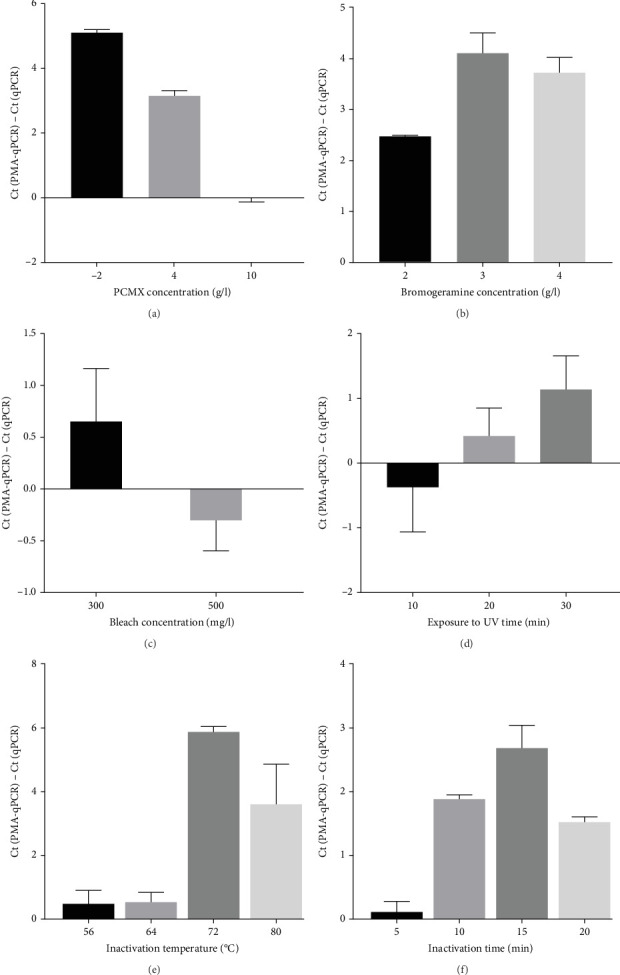
The efficacies of PRRSV inactivation by disinfectants, UV light, and heat were evaluated. The same amount of HP-PRRSV-2 XJ17-5 isolate (200 μl 103 TCID_50_/ml) was used for all inactivation evaluations. The *Δ*Cts (Ct (PMA-qPCR) − Ct(qPCR)) was determined to assess inactivation efficacies. (A) PRRSV inactivation effects by PCMX. (B) PRRSV inactivation effects by bromogeramine. (C) PRRSV inactivation effects by bleach. (D) PRRSV inactivation effects by exposure to UV light for 10–30 min. (E) PRRSV inactivation effects by heat from 56°C to 80°C. (F) PRRSV inactivation effects by heat at 72°C for 5–20 min. Ct, cycle threshold; PCMX, parachlormetaxylenol; PMA, propidium monoazide; PRRSV, porcine reproductive and respiratory syndrome virus; UV, ultraviolet.

**Figure 2:**
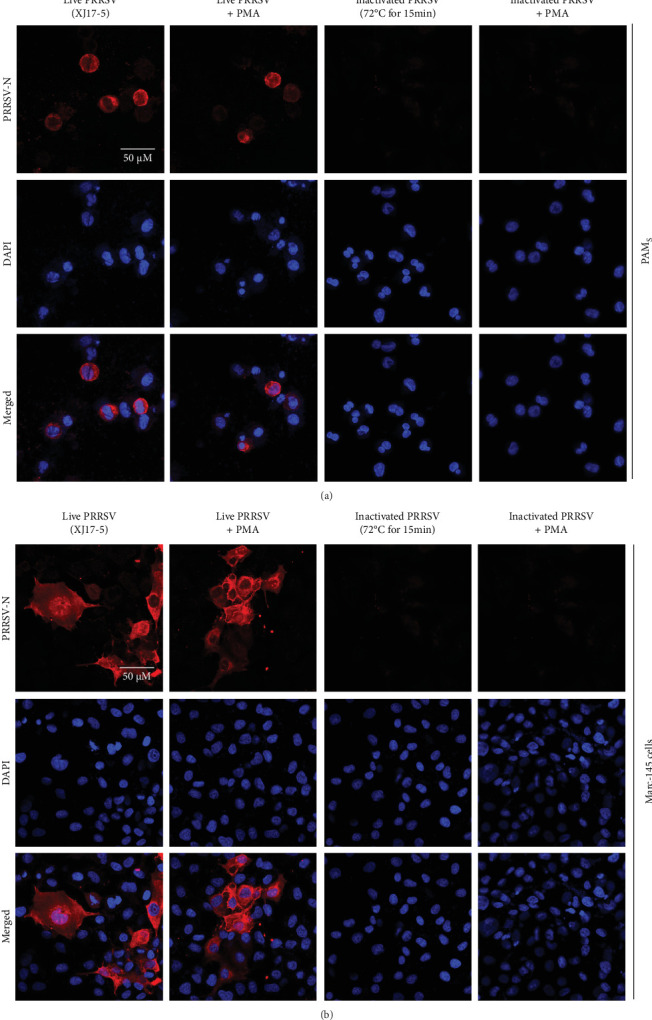
The inactivation efficacy was confirmed by cell culture and viral antigen detection. (A) PAMs were used for infections by live or inactivated (72°C 15 min) XJ17-5 isolates (10^3^ TCID_50_/ml) that were pretreated with or without PMA and then examined by IFA at 72 hpi. (B) Marc-145 cells were used for infections by live or inactivated XJ17-5 isolates pretreating with or without PMA and then examining by IFA at 72 hpi. PRRSV-specific antigen (red signal) could be detected in live XJ17-5 with PMA pretreatment but could not be detected in inactivated XJ17-5 with PMA pretreatment. Live XJ17-5 without PMA pretreatment was set as a positive control. Inactivated XJ17-5 without PMA pretreatment was set as negative control. DAPI, 4′′,6-diamidino-2-phenylindole; IFA, immunofluorescence assay; PAMs, Pulmonary alveolar macrophages; PMA, propidium monoazide; PRRSV, porcine reproductive and respiratory syndrome virus.

To select an effective PRRSV inactivation strategy for developing PRRSV PMA-qPCR assay, we compared the inactivation effects of three disinfectants, UV light, and heat. According to previous studies, PMA pretreatment was preliminarily set as incubation with 50 μM PMA for 15 min and then exposure to blue light for 20 min before qPCR amplification [14, 17, 18, 20, 31]. For three disinfectants, the *Δ*Cts (Ct (PMA-qPCR) − Ct (qPCR)) for 2–10 g/l PCMX were around −0.12–5.18 (Figure 1A), for 2–4 g/l bromogeramine were 2.45–4.46 (Figure 1B), for 300–500 mg/l bleach were−0.54–1.22 (Figure 1C), respectively. When exposure to UV light, the *Δ*Cts ranged from −0.85 to 1.68 (Figure 1D). When inactivated by heat (56–80°C), the *Δ*Cts were from 0.33 to 6.01 (Figure 1E), while the highest *Δ*Ct was achieved at 72°C. We further determined the *Δ*Cts of PRRSV inactivation at 72°C for various times (5–20 min), which were from 0.01 to 3.06 (Figure 1F). Overall, the biggest *Δ*Ct was observed at 72°C for 15 min. In addition, IFA detection in both PAMs and Marc-145 cells further confirmed that all PRRSV virions were completely inactivated by heating at 72°C for 15 min (Figure 2). Therefore, we used 72°C 15 min for inactivation treatment in the following optimization of PRRSV PMA-qPCR assay.

### 3.2. Optimization of PMA-qPCR Assay

**Figure 3:**
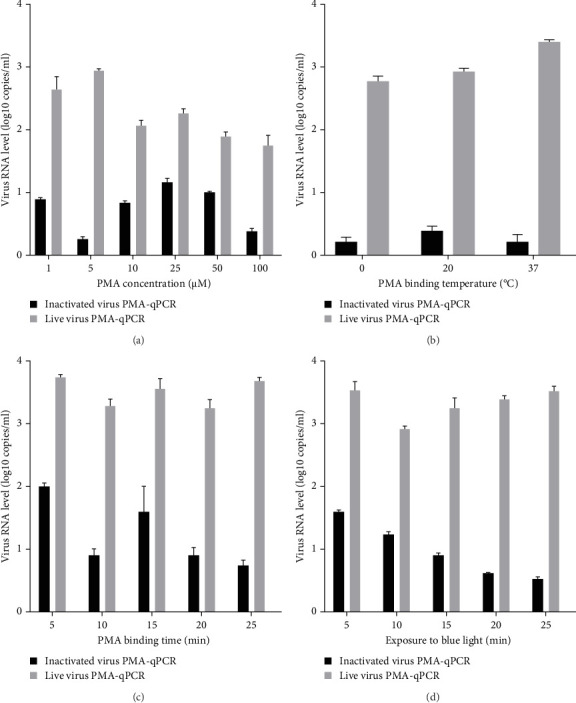
The PMA treatment parameters were optimized. (A) The influence of 1–100 μM PMA pretreatment on PMA-qPCR amplification was determined. (B) The influence of PMA binding temperature (0–37°C) on PMA-qPCR amplification was evaluated. (C) The influence of PMA binding time (5–25 min) on PMA-qPCR amplification was assessed. (D) The influence of photolysis time (exposure to blue light for 5–25 min) on PMA-qPCR amplification was tested. PMA, propidium monoazide.

Live HP-PRRSV-2 XJ17-5 isolate (200 μl 10^3^ TCID_50_/ml) or inactivated XJ17-5 (72°C for 15 min) was used for the optimization of PRRSV PMA-qPCR assay. As shown in Figure 3A, the viral RNA level was the highest for live virus, meanwhile the lowest for inactivated virus when 5 μM PMA was used. The best PMA binding temperature was at 37°C (Figure 3B). Meanwhile, the most suitable PMA binding time and photolysis time were both 25 min (Figure 3C, D). Therefore, the optimized PMA treatment was set as using 5 μM PMA to interact with PRRSV at 37°C for 25 min and then submitting to photolysis with blue light for 25 min.

**Figure 4:**
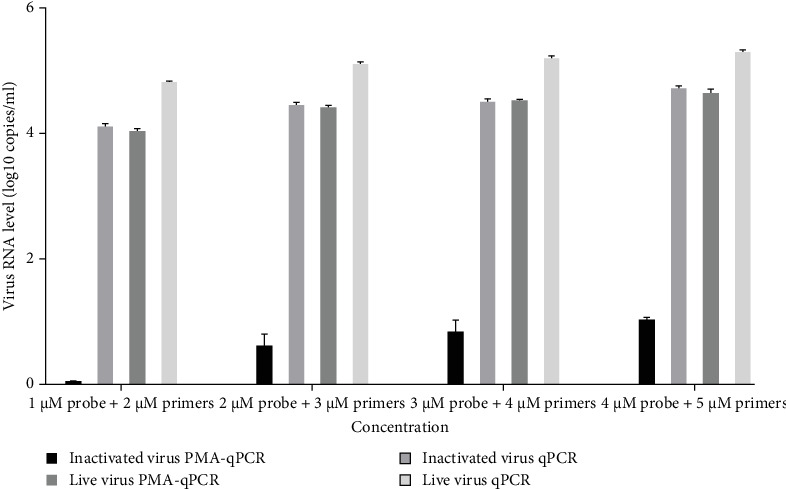
The influence of primers and probe concentrations on PMA-qPCR amplification was determined. When the inactivated virus (72°C for 15 min) was pretreated with PMA, viral RNA was undetectable using 1 μM probe and 2 μM primers, while the other three groups had similar amplification effects at different concentrations of probe and primers. PMA, propidium monoazide.

To optimize the primers and probe concentration, 1–4 μM probe and 2–5 μM primers were used for PRRSV PMA-qPCR tests. As shown in Figure 4, when the inactivated virus (72°C for 15 min) was pretreated with PMA, viral RNA was undetectable using 1 μM probe and 2 μM primers, while the other three groups (inactivated virus without PMA, live virus with PMA, and live virus without PMA) have similar amplification efficacies at different concentrations of probe and primers.

### 3.3. Evaluation of This Newly Developed PRRSV PMA-qPCR Assay

**Figure 5:**
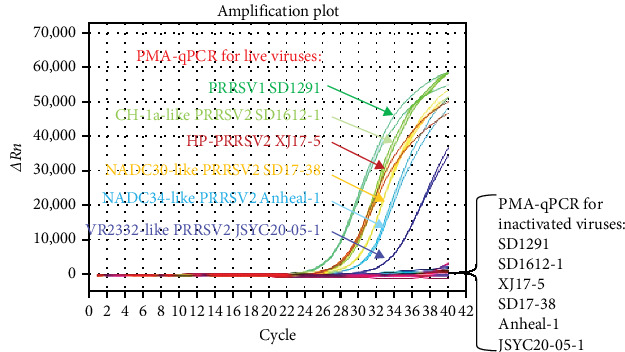
Detection spectrum of the universal PRRSV PMA-qPCR assay. All live PRRSV strains including PRRSV-1 (SD1291 isolate), CH-1a-like PRRSV-2 (SD1612-1 isolate), HP-PRRSV-2 (XJ17-5 isolate), NADC30-like PRRSV-2 (SD17-38 isolate), NADC34-like PRRSV-2 (Anheal-1 isolate), and VR-2332-like PRRSV-2 (JSYC20-05-1 isolate) were detectable while all the corresponding inactivated viruses were undetectable by this PRRSV PMA-qPCR assay. PMA, propidium monoazide; PRRSV, porcine reproductive and respiratory syndrome virus.

To evaluate the detection spectrum of this new PMA-qPCR assay, distinct live and inactivated PRRSV isolates (10^1.2^–10^3.5^ TCID_50_/ml) were tested. As shown in Figure 5, all live viruses including PRRSV-1 (SD1291 isolate), CH-1a-like PRRSV-2 (SD1612-1 isolate), HP-PRRSV-2 (XJ17-5 isolate), NADC30-like PRRSV-2 (SD17-38 isolate), NADC34-like PRRSV-2 (Anheal-1 isolate), and VR-2332-like PRRSV-2 (JSYC20-05-1 isolate) could be detected by PMA-qPCR while all the corresponding inactivated viruses were undetectable. Specificity evaluation confirmed that other common swine viruses could not be detected in this PMA-qPCR assay (Supporting Information: Figure S1A). In addition, this PMA-qPCR has the same sensitivity as qPCR and 10 folds higher sensitivity than the conventional PCR assay (Supporting Information: Figure S1B) [25]. Furthermore, the coefficients of variation (CVs) for intra-assay ranged from 0.17% to 2.37%, and CVs for interassay varied from 1.02% to 2.93% (Supporting Information: Table S1), supporting the satisfied reproducibility of this PMA-qPCR assay.

To further assess the discrimination ability of the PMA-qPCR assay, the newly developed PMA-qPCR was applied to detect serial mixtures of different ratios of live and inactivated viruses. As shown in Table 1, the Ct values generated by qPCR detection on four samples (100% live virus, 10% live virus + 90% inactivated virus, 1% live virus + 99% inactivated virus, 100% inactivated virus) were very close. When detected by PMA-qPCR, the ΔCt values between qPCR and PMA-qPCR on 100% live virus sample, 10% live virus + 90% inactivated virus, and 1% live virus + 99% inactivated virus were 0.39, 3.22, and 6.73, respectively, which were close to the ideal situations. In addition, 100% inactivated virus sample was detected as negative in the PMA-qPCR assay. These results further supported that this PRRSV universal PMA-qPCR assay is able to differentiate live and inactivated PRRSV.

**Figure 6:**
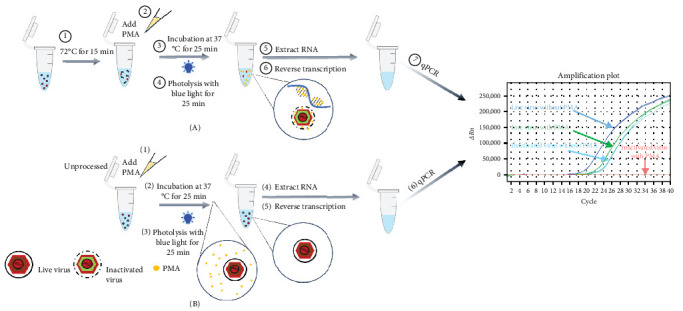
The procedures of the universal PRRSV PMA-qPCR assay. (A) The procedures of PMA-qPCR for inactivated PRRSV isolates. (B) The procedures of PMA-qPCR for live PRRSV or unknown samples. PMA, propidium monoazide; PRRSV, porcine reproductive and respiratory syndrome virus.

To mimic the application of the PMA-qPCR assay on clinical sample detection, serial diluted (10^2.6^–10^0.6^) live or inactivated XJ17-5 viruses were added to negative fecal samples and detected by the PMA-qPCR assay. As shown in Supporting Information: Figure S2, only the serial diluted live viruses could be detected, while serial diluted inactivated viruses were undetectable. Overall, all the above results supported that a PMA-qPCR assay for rapid and universal detection of infectious PRRSV was successfully developed in this study (Figure 6).

### 3.4. Application of PMA-qPCR and qPCR on Clinical Sample Detection

To further evaluate the newly developed PMA-qPCR, 270 clinical samples were submitted to both PMA-qPCR and qPCR evaluation (Table 2). For environmental feces samples, 34 samples (34/57, 59.65%) were detected as PRRSV positive by qPCR, while 23 samples (23/57, 40.35%) were determined positive by PMA-qPCR. There were 30 lungs (30/177, 16.95%) were PRRSV positive in both qPCR and PMA-qPCR. Thirteen LN samples (13/33, 39.39%) were qPCR positive, but only 7 LN samples were PMA-qPCR positive. All three sera were both qPCR and PMA-qPCR positive (Table 3). All 17 qPCR-positive but PMA-qPCR-negative samples were submitted to PRRSV isolations in PAMs, but no virus was successfully isolated. Meanwhile, PRRSV could be successfully isolated in representative PMA-qPCR-positive clinical samples (Supporting Information: Figure S3). These results supported that the newly developed PMA-qPCR could be utilized for live and inactivated PRRSV discrimination in clinical samples.

## 4. Discussion

PRRSV is one of the most economically important swine diseases in the world. In the United States, the estimated cost of PRRS losses in national breeding and growing pig herds is at $664 million annually. The additional costs attributed to PRRS for veterinary, biosecurity, and other outbreak-related costs are estimated to be $477.79 million annually [32]. In China, PRRS causes the economic losses of ~ ¥24 billion, according to statistics of the National Quarantine Department. PRRSV not only could be transmitted by direct contact, but also by contaminated instruments, even by airborne transmission [33]. Rapid detection of PRRSV is mainly relied on molecular biological methods, such as PCR and qPCR [12], and serological method, such as ELISA [34]. However, none of them can distinguish between infectious and noninfectious viruses. Environmental feces containing PRRSV nucleic acid would be detected as PRRSV positive by PCR/qPCR even if the virus was already inactivated by disinfectants, leading to a misdiagnosis of environmental contamination situation (a false positive). In addition, PRRSV-positive tissue or serum samples would not be suitable for virus isolation if the containing virus had already lost its infectivity. However, it is time consuming to determine PRRSV infectivity by traditional live virus detection methods such as virus isolation. Considering the worldwide prevalence and high diversity of PRRSV, a rapid and universal live virus detection assay is essential for effective PRRS diagnosis, prevention, and control.

Viable pathogen tests based on PMA pretreatment have been utilized to detect bacteria and viruses [18, 20, 35]. Viable and dead *Salmonella app*., *Escherichia coli*, and *Staphylococcus aureus* could be simultaneously differentiated by a method combining PMA with multiplex qPCR [35]. Infectious SARS-CoV-2 in PCR-positive samples could be rapidly determined by SDS-PMA-assisted RT-qPCR [17]. However, this method did not really discriminate live and dead SARS-CoV-2 but predicting based on a cutoff *Δ*Ct value (set at 8.6) between PMA-treated and PMA-free groups. PMA-based qPCR assays were established and utilized to evaluate the disinfection effectiveness and infectivity of the African swine fever virus (ASFV) [20, 36]. However, the disinfection efficacy and PMA pretreatment strategy of these two ASFV PMA-qPCR assays were inconsistent. All these previous studies indicated that disinfection methods and PMA pretreatment conditions should be optimized for each individual virus [20]. Here, we developed a cultured-free, PMA-based qPCR assay for rapid and universal detection of infectious PRRSV, which will facilitate better monitor and control of PRRSV transmission.

In this study, we first selected an effective strategy for PRRSV inactivation. Our results showed that all three inactivation strategies (disinfectants [PCMX, bromogeramine, and bleach], UV light, and heat) may inactivate PRRSV in a certain degree. However, high concentrations of PCMX (10 g/l) and bleach (500 mg/l) may affect the following PMA pretreatment and PCR amplification. Therefore, we would recommend diluting the clinical samples if high-concentration disinfectants were used for viral inactivation. Even though heat inactivation is not suitable for ASFV inactivation [20], our results showed that heat at 72°C for 15 min is an effective PRRSV inactivation strategy, which was further confirmed by virus isolation and IFA detection. The different inactivation efficacy is probably due to the significant architectural difference between ASFV and PRRSV virions [33, 37].

Previous studies indicated that 4–25 μM PMA is generally utilized for bacteria, while 50–150 μM PMA is commonly used to treat viruses [17, 18, 20, 36]. For instance, 30 μM PMA was selected to differentiate viable and dead *Salmonella app*., *E. coli*, and *S. aureus* [35], 50 μM PMA pretreatment was utilized to determine infectious SARS-CoV-2 in PCR-positive samples [17], while 100 μM PMA was used to distinguish infectious and noninfectious enteric viruses in water samples [14]. However, our results showed that 5 μM PMA not only did not influence PCR amplification (highest live virus amplification) but also could most effectively discriminate inactivated virus (lowest inactivated virus amplification). In addition, PMA binding temperature, PMA binding time, and exposure time to blue light also influence the PMA pretreatment effects at certain levels. Therefore, all these parameters should be optimized term-by-term for each virus-specific PMA-qPCR assay.

In the aspect of determining PRRSV infectivity, this universal PRRSV PMA-qPCR assay has some advantages than the gold standard method of virus isolation. First, it is more convenient to execute universal PRRSV PMA-qPCR for detecting PRRSV infectivity, which may be applied in quality monitoring of PRRS-killed vaccine. Meanwhile, it might also help detecting and comparing virus titers in PRRS MLV vaccines. Second, it could assess the presence of infectious PRRSV in positive samples within 2 h, which highly facilitated the selection of PRRSV-positive samples for virus isolation in the laboratories. Third, it is developed using primers and probes that could detect all prevalent PRRSV isolates [25], ensuring this PMA-qPCR is also able to detect currently prevalent PRRSV-1 and PRRSV-2 isolates. More importantly, the application and comparison of PMA-qPCR and qPCR assays on environmental samples and pig samples provided evidence that our PMA-qPCR assay might not only assist in more rapid and accurate determination on PRRSV infectivity of pig samples but also PRRSV environmental contamination situation in pig farms.

In conclusion, this is the first report of PRRSV PMA-qPCR assay for discriminating infectious and noninfectious PRRSV isolates. The optimized PRRSV PMA-qPCR assay not only can be applied in the laboratory for clinical positive sample selection but also can be used for PRRS vaccine quality monitoring. More importantly, it may be utilized for environmental contamination monitoring. Overall, this study provides an effective alternative strategy for infectious PRRSV determination.

## Figures and Tables

**Table 1:** PMA-qPCR detection on serial mixtures of live and inactivated viruses.

Group	100% live virus	10% live virus + 90% inactivated virus	1% live virus + 99% inactivated virus	100% inactivated virus
qPCR	26.43 ± 0.38	26.13 ± 0.07	26.52 ± 0.30	26.53 ± 0.35
PMA-qPCR	26.82 ± 0.30	29.35 ± 0.64	33.25 ± 0.42	/^#^
*Δ*Ct	0.39 (0)*⁣*^*∗*^	3.22 (3.33)	6.73 (6.67)	/

Abbreviations: Ct, cycle threshold; PMA, propidium monoazide.

*⁣* ^*∗*^The ideal *Δ*Ct values between qPCR and PMA-qPCR were shown in the brackets.

^#^The slash indicates undetectable.

**Table 2:** Clinical sample information.

Sample type	Sample no.	Sample percentage (%)	PRRSV positive no.	Positive percentage (%)
Feces	57	21.11	34	59.65
Lung	177	65.56	30	16.95
LN	33	12.22	13	39.39
Serum	3	1.11	3	100
Total	270	100	80	29.63

Abbreviation: LN, lymph nodes.

**Table 3:** Comparison of PMA-qPCR and qPCR detection on PRRSV-positive clinical samples.

No.	Name	Sample	Ct		No.	Name	Sample	Ct	
			qPCR	PMA-qPCR				qPCR	PMA-qPCR
1	JSNJ20-869	Feces	26 ± 0.24	ND	41	SCNJ23-1875	Lung	25.82 ± 0.05	27.58 ± 0.19
2	JSNJ20-873	Feces	31.34 ± 0.92	ND	42	HNZK23-1963	Lung	27.41 ± 0.01	27.25 ± 0.59
3	FJFZ20-983	Feces	14.9 ± 0.16	23.51 ± 0.21	43	HNZK23-1971	Lung	27.43 ± 0.20	27.33 ± 0.29
4	FJFZ20-984	Feces	18.34 ± 0.03	24.49 ± 0.03	44	HNZK23-1972	Lung	27.71 ± 0.31	27.92 ± 0.05
5	FJFZ20-985	Feces	15.7 ± 0.21	23.62 ± 0.03	45	JSYZ23-2577	Lung	25.31 ± 0.13	24.9 ± 0.12
6	FJFZ20-986	Feces	15.72 ± 0.15	20.45 ± 0.14	46	JSYZ23-2582	Lung	27.62 ± 0.03	27.66 ± 0.30
7	FJFZ20-987	Feces	15.27 ± 0.26	20.57 ± 0.09	47	BJ23-2652	Lung	24 ± 0.23	24.36 ± 0.25
8	FJFZ20-988	Feces	19.49 ± 0.03	23.98 ± 0.08	48	BJ23-2663	Lung	23.89 ± 0.41	25.38 ± 0.31
9	FJFZ20-995	Feces	26.85 ± 0.02	30.36 ± 0.38	49	JSYZ23-2667	Lung	20.34 ± 0.19	22.16 ± 0.09
10	FJFZ20-996	Feces	31.09 ± 0.06	ND	50	HLJSYS23-1656	Lung	29.15 ± 0.24	29.46 ± 0.23
11	FJFZ20-997	Feces	30.39 ± 0.22	ND	51	HLJSYS23-1661	Lung	28.82 ± 0.64	29.33 ± 0.67
12	FJFZ20-998	Feces	31.78 ± 0.27	ND	52	SDLY23-1733	Lung	28.76 ± 0.29	29.56 ± 0.60
13	FJFZ20-993	Feces	19.77 ± 0.04	25.52 ± 0.01	53	SDLY23-1734	Lung	21.38 ± 0.56	24.38 ± 0.81
14	FJFZ20-994	Feces	20.11 ± 0.98	27.19 ± 0.57	54	SDLY23-1735	Lung	23.84 ± 0.68	28.14 ± 0.53
15	FJFZ20-999	Feces	29.14 ± 0.94	29.08 ± 0.08	55	SDLY23-1736	Lung	23.92 ± 0.89	24.17 ± 0.76
16	FJFZ20-1000	Feces	28.79 ± 0.31	30.57 ± 0.05	56	SDLY23-1742	Lung	15.5 ± 0.20	20.19 ± 0.77
17	FJFZ20-1026	Feces	22.24 ± 0.07	26.58 ± 0.43	57	SDLY23-1744	Lung	23.42 ± 0.31	27.24 ± 0.29
18	FJFZ20-1027	Feces	29.54 ± 0.27	ND	58	JSYZ23-2669	Lung	22.62 ± 0.67	22.08 ± 0.25
19	SDZB20-1047	Feces	27.57 ± 0.01	28.46 ± 0.05	59	JSYZ23-2670	Lung	15.94 ± 0.60	20.92 ± 0.53
20	SDZB20-1049	Feces	22.83 ± 0.41	24.9 ± 0.07	60	JSYZ23-2668	Lung	21.53 ± 0.15	24.84 ± 0.12
21	SDZB20-1050	Feces	28.55 ± 0.15	28.48 ± 0.22	61	HLJSYS23-1648	Lung	28.19 ± 0.31	28.22 ± 0.07
22	SDZB20-1051	Feces	28.67 ± 0.05	31.92 ± 0.66	62	JSYZ23-2582	Lung	27.62 ± 0.41	27.66 ± 0.29
23	SDZB20-1052	Feces	27.9 ± 0.30	30.46 ± 0.39	63	JSYZ24-2708	Lung	20.92 ± 0.07	24.65 ± 0.12
24	SDZB20-1054	Feces	27.26 ± 0.47	29.4 ± 0.15	64	JSYZ24-2709	Lung	16.76 ± 0.07	16.83 ± 0.01
25	SDZB20-1055	Feces	28.59 ± 0.35	29.23 ± 0.03	65	GDCZ23-2333	LN	26.61 ± 0.28	26.6 ± 0.25
26	SDZB20-1057	Feces	28.23 ± 0.03	29.44 ± 0.09	66	GDCZ23-2338	LN	26.61 ± 0.32	27.48 ± 0.39
27	SDZB20-1058	Feces	25.58 ± 0.30	26.98 ± 0.14	67	GDCZ23-2339	LN	30.95 ± 0.01	ND
28	SDZB20-1060	Feces	28.88 ± 0.23	ND	68	GDCZ23-2340	LN	27.21 ± 0.40	27.47 ± 0.39
29	SDZB20-1061	Feces	25.81 ± 0.09	30.04 ± 0.12	69	GDCZ23-2341	LN	27.25 ± 0.21	27.42 ± 0.29
30	SDZB20-1062	Feces	27.83 ± 0.09	30.01 ± 0.16	70	GDCZ23-2348	LN	32.4 ± 0.30	ND
31	SDZB20-1063	Feces	29.35 ± 0.21	ND	71	GDCZ23-2346	LN	28.12 ± 0.01	ND
32	SDZB20-1064	Feces	28.98 ± 0.19	ND	72	GDCZ23-2349	LN	28.11 ± 0.01	ND
33	SDZB20-1065	Feces	25.99 ± 0.14	ND	73	GDCZ23-2350	LN	28.41 ± 0.08	ND
34	JSYC21-1245	Feces	31 ± 0.78	ND	74	GDCZ23-2466	LN	27.3 ± 0.16	27.01 ± 0.19
35	SDLY23-1737	Lung	27.71 ± 0.33	27.52 ± 0.21	75	GDCZ23-2462	LN	27.4 ± 0.11	27.62 ± 0.01
36	SDLY23-1738	Lung	24.87 ± 0.09	26.76 ± 0.15	76	GDCZ23-2637	LN	21.26 ± 0.21	23.97 ± 0.48
37	SDLY23-1739	Lung	25.96 ± 0.32	27.58 ± 0.55	77	GDCZ23-2645	LN	32.21 ± 0.28	ND
38	SDLY23-1740	Lung	23.56 ± 0.07	25.35 ± 0.01	78	GDCZ23-2122	Serum	28.65 ± 0.57	30.31 ± 0.85
39	SDLY23-1741	Lung	26.32 ± 0.23	27.17 ± 0.19	79	JSYZ24-2706	Serum	31.59 ± 0.31	29.64 ± 0.21
40	SDLY23-1743	Lung	24.28 ± 014	27.45 ± 0.10	80	JSYZ24-2707	Serum	31.59 ± 0.17	29.64 ± 0.72

Abbrevaitions: Ct, cycle threshold; LN, lymph nodes; ND, not detectable; PMA, propidium monoazide; PRRSV, porcine reproductive and respiratory syndrome virus.

## Data Availability

The data used to support the findings of this study are included within the article and supporting files.

## Supporting Information

Supporting Information Figure S1. Specificity and sensitivity evaluation of PRRSV PMA-qPCR assay. (A) FAM fluorescent signal was observed when PRRSV (XJ17-5 isolate) was detected but not for other viruses. (B) The detection limit of PRRSV PMA-qPCR assay is 100.3 TCID_50_/ml, which is 10 times higher than conventional PCR assay and the same as PRRSV qPCR assay [25]. Figure S2. Evaluation of PRRSV PMA-qPCR assay on mimic clinical samples. Serial diluted (102.6 100.6) live or inactivated XJ17-5 viruses were added to negative fecal samples and detected by PRRSV universal PMA-qPCR assay. Only the serial diluted live viruses could be detected, while serial diluted inactivated viruses could not be detected. Figure S3. PRRSV isolations from representative clinical samples. Three qPCR and PMA-qPCR positive samples and three qPCR positive but PMA-qPCR negative samples (100 μl filtered supernatant from each sample homogenate) were selected and used for virus isolation in PAMs. IFA detection was performed at 72 hpi. PRRSV N protein-specific signals could only be detected in three qPCR and PMA-qPCR positive samples. XJ17-5 infection (0.01 MOI) was set as a positive control, and RPMI-1640 was added to a negative control. Table S1. Intrarepeatability and interreproducibility of this PRRSV PMA-qPCR assay.
