# Supplementary material for: Propidium Monoazide Integrated With qPCR Enables Rapid and Universal Detection of Infectious Porcine Reproductive and Respiratory Syndrome Viruses
Source: Transbound Emerg Dis. 2024 Dec 21;2024:6250851. doi: 10.1155/tbed/6250851 (PMC12017026; doi:10.1155/tbed/6250851)
Supplement: Supporting Information — Figure S1. Specificity and sensitivity evaluation of PRRSV PMA-qPCR assay. (A) FAM fluorescent signal was observed when PRRSV (XJ17-5 isolate) was detected but not for other viruses. (B) The detection limit of PRRSV PMA-qPCR assay is 100.3 TCID50/ml, which is 10 times higher than conventional PCR assay and the same as PRRSV qPCR assay [25]. Figure S2. Evaluation of PRRSV PMA-qPCR assay on mimic clinical samples. Serial diluted (102.6 100.6) live or inactivated XJ17-5 viruses were added to negative fecal samples and detected by PRRSV universal PMA-qPCR assay. Only the serial diluted live viruses could be detected, while serial diluted inactivated viruses could not be detected. Figure S3. PRRSV isolations from representative clinical samples. Three qPCR and PMA-qPCR positive samples and three qPCR positive but PMA-qPCR negative samples (100 μl filtered supernatant from each sample homogenate) were selected and used for virus isolation in PAMs. IFA detection was performed at 72 hpi. PRRSV N protein-specific signals could only be detected in three qPCR and PMA-qPCR positive samples. XJ17-5 infection (0.01 MOI) was set as a positive control, and RPMI-1640 was added to a negative control. Table S1. Intrarepeatability and interreproducibility of this PRRSV PMA-qPCR assay. [file 6250851.f1.pdf]

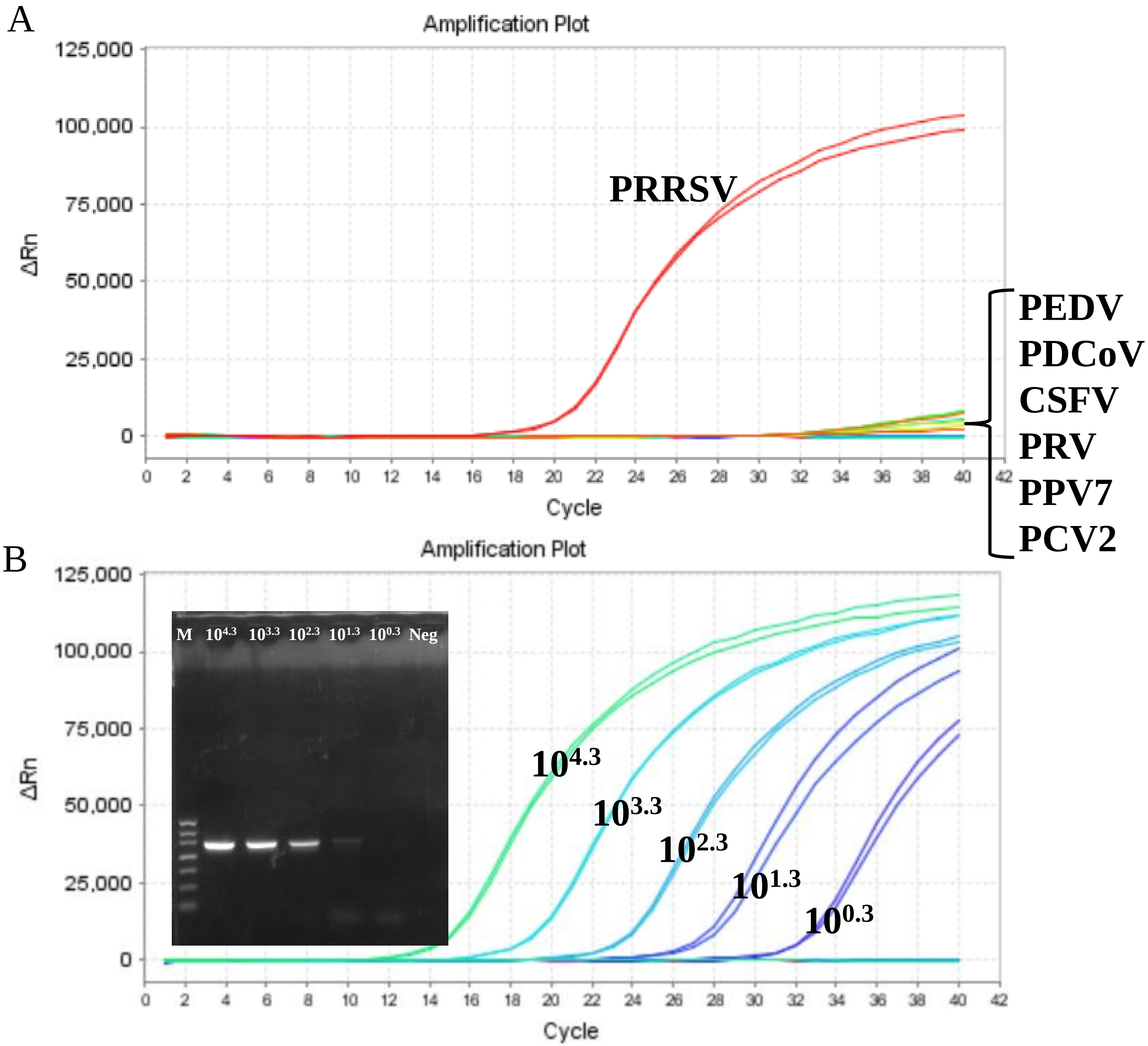

Figure S1. Specificity and sensitivity evaluation of PRRSV PMA-qPCR assay. (A) FAM fluorescent signal was observed when PRRSV (XJ17-5 isolate) was detected but not for other viruses. (B) The detection limit of PRRSV PMA-qPCR assay is $10^{0.3}$ TCID50/ml, which is 10 times higher than conventional PCR assay and same as PRRSV qPCR assay (Chen et al., 2019b).

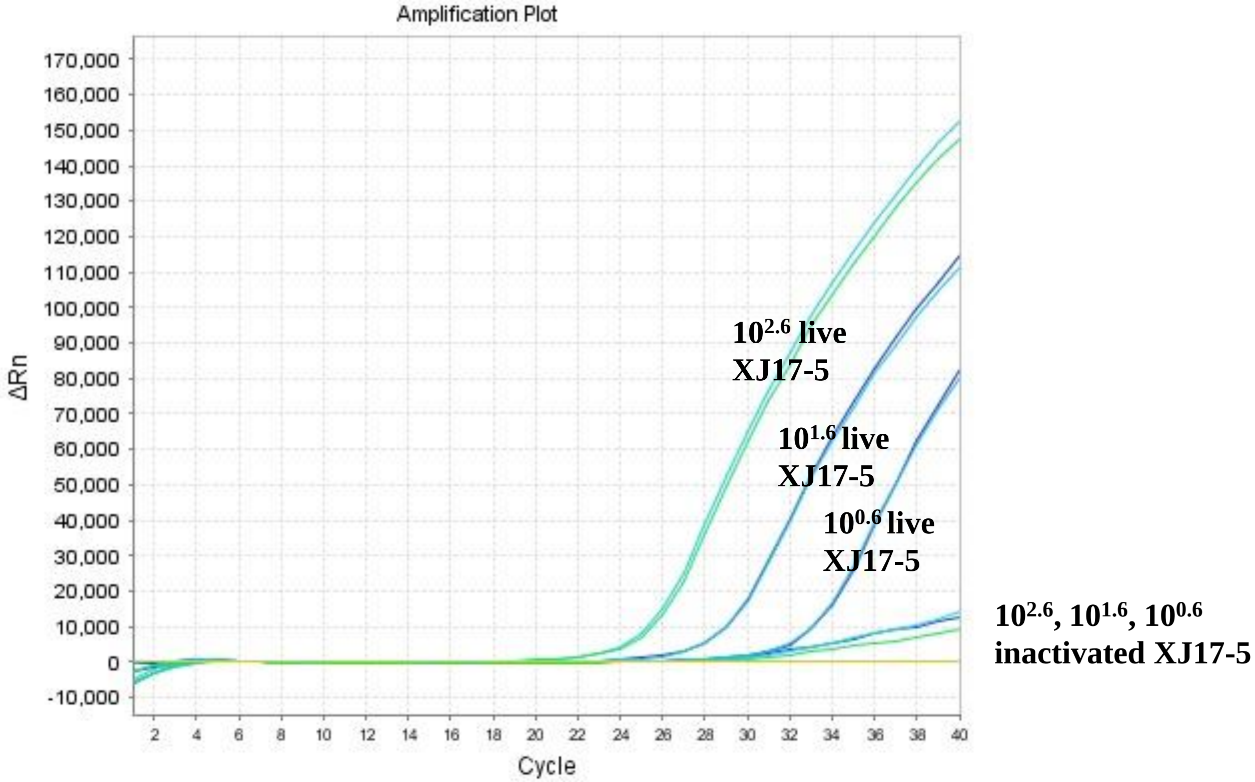

Figure S2. Evaluation of PRRSV PMA-qPCR assay on mimic clinical samples. Serial diluted ($10^{2.6}$ ~ $10^{0.6}$) live or inactivated XJ17-5 viruses were added to negative fecal samples and detected by PRRSV universal PMA-qPCR assay. Only the serial diluted live viruses could be detected while serial diluted inactivated viruses could not be detected.

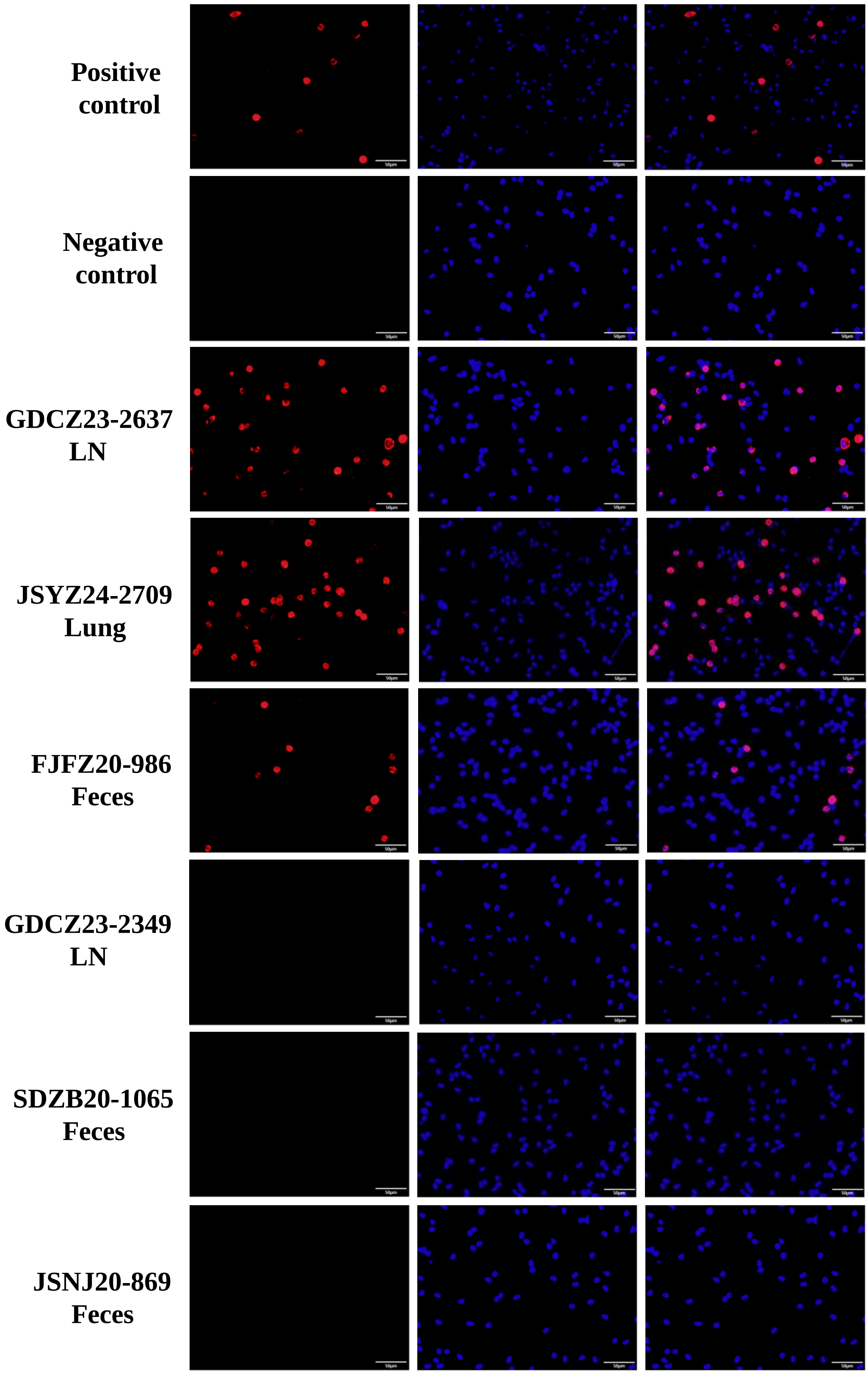

Figure S3. PRRSV isolation from representative clinical samples. Three qPCR and PMA-qPCR positive samples and three qPCR positive but PMA-qPCR negative samples (100 µl filtered supernatant from each sample homogenate) were selected and used for virus isolation in PAMs. IFA detection was performed at 72 hpi. PRRSV N protein specific signals could only be detected in three qPCR and PMA-qPCR positive samples. XJ17-5 infection (0.01 MOI) was set as positive control and RPMI-1640 was added to negative control.

Table S1. Intra-repeatability and inter-reproducibility of this PRRSV PMA-qPCR assay

Sample *	Intra-repeatability	Coefficient of variation (CV in %)	Inter-reproducibility	Coefficient of variation (CV in %)
10^5 $TCID_{50}$/ml	15.73+0.04 #	0.17	15.754±0.41	1.02
10^3 $TCID_{50}$/ml	20.84±0.57	2.37	21.25±0.815	2.93
10^1 $TCID_{50}$/ml	27.13±0.27	1.65	27.38±0.75	2.71

* Different amounts (10^1 ~ 10^5 $TCID_{50}$/ml) of HP-PRRSV2 XJ17-5 isolate was used.

# The mean of Ct ±standard deviation from five replicates.
